# In Memoriam—Dr. Mahlon Preston

**Published:** 1895-11

**Authors:** W. A. D. Pierce

**Affiliations:** Corresponding Secretary; Philadelphia


### IN MEMORIAM—DR. MAHLON PRESTON.

### Meeting of the Homœopathic Medical Council.

Philadelphia, October 16th, 1895.

The Council was called to order by the President, Dr. Levi Hoopes.

Dr. W. A. D. Pierce, seconded by Dr. Jesse Thatcher, moved that the regular business be suspended, and the Council proceed to adopt resolutions on the death of Mahlon Preston, M. D., of Norristown, which resolution was adopted unanimously.

Dr. Pierce read letters of regret for absence from Drs. Walter M. James and Frederick Preston. Dr. Preston's letter also said, "I wish to thank you and other members of the Council who kindly showed their sympathy for my brother by visiting him," etc. * * * "In this connection it would be impossible to refrain from mentioning his attending physician, Dr. Walter M. James, whose earnestness and never-tiring zeal for his patient through his long illness of nearly a year, imperatively demand public recognition at a session of this Society.

"Myself, and my brother's family, desire that Dr. James and all our acquaintances should know that we consider his services and true kindness past all recompense.

"I personally believe that at one point the Doctor came very near to achieving success in a case which seemed absolutely hopeless from the first."

On motion of Dr. E. A. Krusen, seconded by Dr. Samuel Long, a committee of three was appointed to draft resolutions, etc., on account of the death of Dr. Mahlon Preston. Dr. E. A. Krusen, Dr. W. A. D. Pierce, and Dr. Samuel Long were the committee. The following are the resolutions as reported and accepted :

WHEREAS, It has pleased our Heavenly Father, in His all-wise Providence, to remove from our midst by death our beloved brother, Mahlon Preston, M. D., and,

WHEREAS, We know of the untiring zeal and regular attendance of his physicians, Drs. Walter M. James and Frederick Preston, during his long illness. Therefore be it

*Resolved*, That we deeply feel and sincerely regret the loss of him from our ranks. We humbly bow in submission to the will of Him who doeth all things well.

*Resolved*, That the Homœopathic Medical Council has lost a true and faithful member, his family a noble husband and father, and the community a valuable citizen.

*Resolved*, That we hereby extend our heartfelt sympathy to his bereaved wife and family in this, their great affliction.

*Resolved*, That the Homœopathic Medical Council, which was founded in the year 1881 by our esteemed friend, acknowledge his superior judgment and skill as a physician, and that the community in which he labored, will only in time appreciate their loss.

*Resolved*, That we commend the eminent services of our fellow-members, Drs. Walter M. James and Frederick Preston, during the long sickness of our departed brother.

*Resolved*, That a copy of these resolutions be sent to his family, that they be extended upon the minutes of this Council, and that copies be furnished for publication to THE HOMŒOPATHIC PHYSICIAN and *The Norristown Herald*.

Dr. Long addressed the meeting on the "Value of the Council." Said he would never voluntarily leave the society, he received benefits at every meeting from the proceedings, and that he hoped the society would be perpetuated as a memorial of its founder, Dr. Mahlon Preston, and that "the memory of our late friend, would help to hold us together."

Dr. Thatcher and others offered similar thoughts and the Council proceeded to regular business.

After presentation of a case for discussion by Dr. Levi Hoopes the Council adjourned, to meet again at the Bullitt Building, November 20th, 1895.

W. A. D. PIERCE, M. D., *Corresponding Secretary.*

